# miR-511 and miR-1297 Inhibit Human Lung Adenocarcinoma Cell Proliferation by Targeting Oncogene *TRIB2*

**DOI:** 10.1371/journal.pone.0046090

**Published:** 2012-10-05

**Authors:** Chao Zhang, Yong Liang Chi, Ping Yu Wang, Ya Qi Wang, Yan Xia Zhang, Jingti Deng, Chang Jun Lv, Shu Yang Xie

**Affiliations:** 1 Key Laboratory of Tumour Molecular Biology in Binzhou Medical University, Department of Biochemistry and Molecular Biology, Binzhou Medical University, YanTai, ShanDong, P.R.China; 2 Shandong China Traditional Medical Affiliated Hospital, Ji Nan, P.R.China; 3 Department of Biochemistry and Molecular Biology, School of Medicine, Shandong University, Ji'nan, P.R.China; 4 The Affiliated Hospital to Binzhou Medical University, BinZhou, P.R.China; University of Saarland Medical School, Germany

## Abstract

microRNAs (miRNAs) are small noncoding RNAs that regulate genes and contribute to many kinds of human diseases, including cancer. Two miRNAs, miR-511 and miR-1297, were investigated for a possible role in adenocarcinoma based on predicted binding sites for the *TRIB2* oncogene by microRNA analysis software, and the pcDNA-GFP-TRIB2–3′UTR vector was constructed to investigate the interaction between *TRIB2* and miR-511/1297 in the adenocarcinoma cell line A549. Green fluorescent protein (GFP) expression was estimated by fluorescence microscopy and flow cytometry after A549 cells were co-transfected with miR-511 (or miR-1297) and pcDNA-GFP-TRIB2–3′UTR vector. The expression of GFP in the miR-511- and miR-1297-treated cells was significantly downregulated in contrast with the negative-control (NC) miRNA-treated cells. The decreased expression of *TRIB2* was further detected after miR-511 (or miR-1297) treatment by western blotting. The MTT test showed inhibition of A549 cell proliferation and Annexin V-FITC/PI dual staining showed increased apoptosis in the miR-511- and miR-1297-treated cells compared to the NC cultures. A transcription factor downstream of *TRIB2*, the CCAAT/enhancer-binding protein alpha (C/EBPα), was expression at higher levels after miR-511 (or miR-1297) decreasing TRIB2 expression. Our results illustrate that miR-511 and miR-1297 act as tumor suppressor genes, which could suppress A549 cell proliferation *in vitro* and *in vivo* by suppressing *TRIB2* and further increasing C/EBPα expression.

## Introduction

Lung cancer is the most common and the leading cause of cancer death in males [1]. Most primary lung cancers, meaning those originating in the lung, are epithelial cell-derived carcinomas. The common symptoms of lung cancer include weight loss, shortness of breath and coughing (may include blood in the sputum). The predominant type of lung cancer is non-small-cell lung cancer (NSCLC), which includes lung adenocarcinoma. The causes of lung cancer are often attributed to a combination of tobacco smoke, genetic factors [2], [3], radon gas [4], and air pollution [5]–[7], and may include other factors. Patients survival depends on cancer stage, general health status of patient, and other factors, and the five-year survival rate is around 14% following diagnosis. The search for biological markers of lung cancer has progressed substantially for use in clinical applications [8]. However, the biological targets for treatment are still largely elusive in lung cancer.

Tribbles was first identified in *Drosophila* as an inhibitor of mitosis that regulates cell proliferation, migration and morphogenesis during development. In mammals, three genes encoding for tribbles homologues have been designed *trib1,trib2 and trib3* [9], [10], and are associated with human malignancies. Recently, TRIB3 has been reported to regulate AKT1 activation in liver by insulin and to regulate ATF4 activity [11], [12]. Several previous studies showed that *TRIB2* acted as a myeloid oncogene and was involved in human leukemia. Strong evidence demonstrated that dysregulated TRIB2 expression contributed to the pathogenesis of acute myeloid leukemia (AML) [13], [14]. *TRIB2* is elevated in a subset of AML patient samples and *TRIB2* has been identified as an oncogene capable of inducing AML in mice by inhibiting the transcription factor C/EBPα [13].

miRNA is a class of 20–22 nt non-coding single-stranded RNA that has been widely found in eukaryotes. It has a variety of biological functions, such as controlling cell differentiation, proliferation and apoptosis [15], by negatively regulating the expression of its targeted genes. Aberrant miRNA expression has been found in many kinds of tumor cells, suggesting that miRNA may be related to tumorigenesis by acting as oncogenes or as tumor suppressor genes via regulation of apoptosis and proliferation of cells. Several miRNAs have been shown to be important in tumorigenesis by downregulating tumor suppressor genes or oncogenes [16], [17]. For instance, it has been demonstrated that miR-1 and miR-133a function as tumor suppressors in prostate cancer by targeting PNP, while miR-21 is involved in cervical squamous cell tumorigenesis by targeting CCL20 [18], [19].

Considering the important roles of miRNAs in controlling cell differentiation as well as the oncogenic role of *TRIB2*, we speculated that TRIB2 expression may be altered by miRNAs and explored *TRIB2-* related miRNAs for lung adenocarcinoma therapy. We predicted the possible miRNAs targeting the 3′-UTR of *TRIB2* using microRNA analysis software and tested their effects on human adenocarcinoma cell apoptosis. Our results demonstrated that the apoptotic rate was increased in the miR-511 (or miR-1297)-treated cells compared with the negative-control miRNA (NC)-treated cells, and these miRNAs could reduce adenocarcinoma cell proliferation by inhibiting TRIB2 expression.

## Results

### High expression of *TRIB2* in lung adenocarcinoma

**Figure 1:**
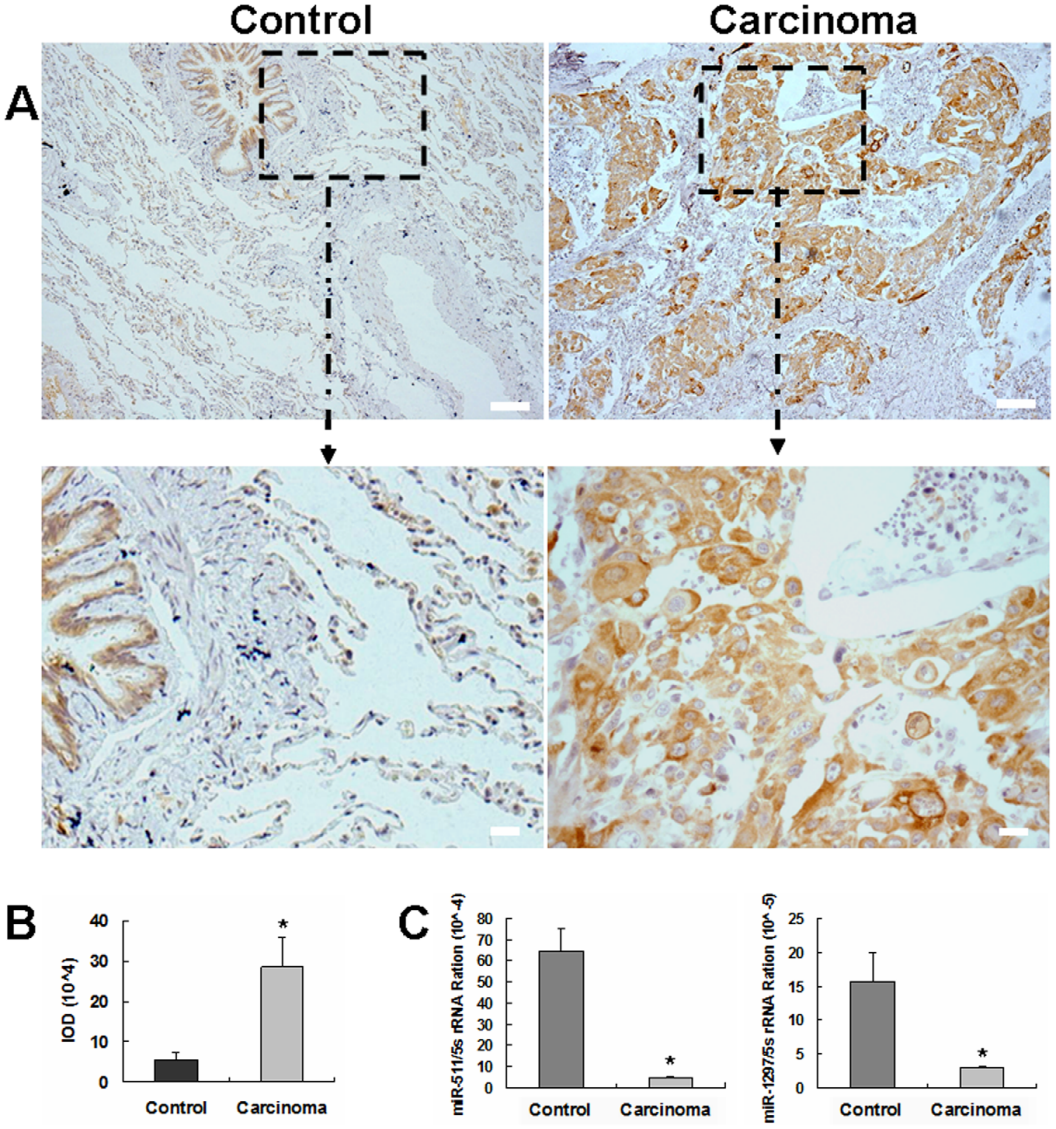
The expression of TRIB2 and miR-511/1297 on control tissue and adenocarcinoma of lung. (**A,B**) Immunohistochemistry and IOD analysis of *TRIB2*. Upper row: Scale bar = 200 µm. Lower row: Scale bar = 20 µm. Control, the para-carcinoma tissues. Carcinoma, adenocarcinoma of lung. TRIB2 expression (Fig. 1 A) and its IOD in the adenocarcinoma tissue (Fig. 1 B) were higher than that of the control tissue (p<0.01). (**C**) Real-time PCR showed that the expression of miR-511 and miR-1297 was much lower in the lung adenocarcinoma than that of control tissue (p<0.05).

*TRIB2* has been identified as an oncogene capable of inducing AML in mice in a previous study [13]. We sought to determine whether *TRIB2* plays an oncogenic role in the tumorigenesis of lung adenocarcinoma. By immunohistochemistry, we observed TRIB2 expression to be higher in human lung adenocarcinoma than in para-carcinoma tissue controls (Figure 1 A B), supporting a possible oncogenic role for *TRIB2* in the pathological changes of lung adenocarcinoma.

### *TRIB2*–3′UTR is regulated by miR-511 and miR-1297

**Figure 2:**
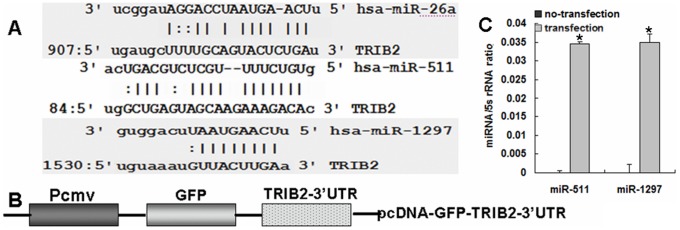
The targeting sites of TRIB2–3′UTR and structural map of the vector. (**A**) The targeting sites on TRIB2–3′UTR. miR-511, miR-1297 and miR-26a are shown. Each miRNA is aligned with the mRNA of human *TRIB2* with the nucleotide position on the *TRIB2* mRNA indicated. Vertical lines indicate identity; gaps indicate mismatch. (**B**) Structural map of the pcDNA-GFP-TRIB2–3′UTR vector. 3′-UTR of *TRIB2* is indicated downstream of the GFP gene. Pcmv: cmv promoter. GFP: green fluorescent protein. *TRIB2*–3′UTR, 3′-UTR of the *TRIB2* gene. (**C**) Real-time PCR detection. 24 h after lung adenocarcinoma cells were treated with miRNAs, higher miR-511 and miR-1297 levels were found in the transfected cells than in the non-transfected cells.

The relationship between the *TRIB2*–3′-UTR and its targeted miRNAs was predicted using microRNA analysis software, which showed that the 3′UTR of *TRIB2* might be targeted by miR-511, miR-1297, et al (Figure 2A), which were not published before. The pcDNA-GFP-TRIB2–3′UTR vector was then constructed (Figure 2B). These miRNAs, negative control, and mutation miRNAs were chemically synthesized in the form of small interfering RNA (siRNA) duplexes according to Park's study (Park SY *et al*., 2009) (Table 1).

**Table 1:** The sequences of chemically synthesized miRNA.^a^

Oligos		Sequence (5′→3′)
miR-511	sense	gugucuuuugcucugcaguca
	antisense	ugacugcagagcaaaagacacuu
miR-1297	sense	uucaaguaauucaggug
	antisense	caccugaauuacuugaauu
miR-26a	sense	uucaaguaauccaggauaggcu
	antisense	agccuauccuggauuacuugaauu
negative control(NC)	sense	caguacuuuuguguaguacaa
	antisense	Guacuacacaaaaguacuguu
mut-miR-511	sense	gu**a**u**ac**uuugcucugcaguca *
	antisense	ugacugcagagcaaaguauacuu
mut-miR-1297	sense	uu**g**a**ua**uaauucaggug *
	antisense	caccugaauuauaucaauu

a The selected miRNAs were chemically synthesized in the form of small interfering RNA (siRNA) duplexes.

* the bold and underlined letters were the mutation sites of miRNA.

**Figure 3:**
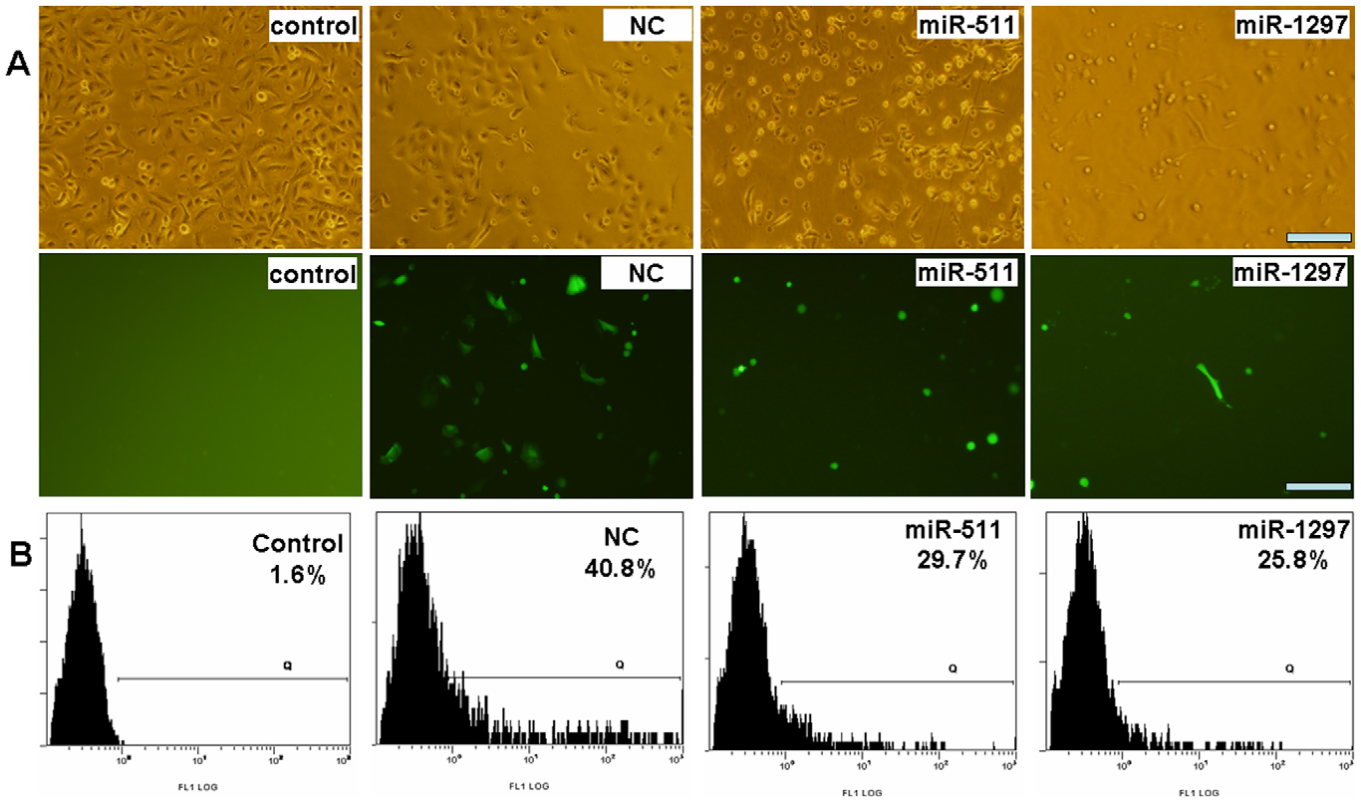
GFP expression detection by fluorescence microscopy and FACS. (**A**) Fluorescence microscopy: Upper panel, phase-contrast view under visible light. Lower panel, fluorescence to reveal expression of GFP-positive cells. Scale bar = 100 µm. The intensity of GFP-positive cells was weaker and the number of GFP-positive cells was fewer in miR-511- or miR-1297-treated cells than that of control cultures. (**B**) FACS results showed that the ratio of GFP-positive cells in miR-511- and miR-1297-treated cultures were much lower than that of NC-treated cells.

After the reporter plasmid (pcDNA-GFP-TRIB2–3′UTR vector) was constructed, miRNA was co-transfected with the reporter plasmid into A549 cells, and higher miRNAs were detected in the miRNA-treated cells than in the untreated cells (Figure 2C). The GFP expression levels were then estimated by examination under fluorescence microscopy and by flow cytometry. The intensities of fluorescence from the miRNA-treated cultures were all decreased and the number of GFP-positive cells was reduced in comparison to the control cultures (Figure 3A), indicating a partial knock-down of expression of the TRIB2-GFP reporter by these miRNA molecules tested. Particularly, the intensity of fluorescence in the miR-511- and miR-1297-treated cells provided the strongest inhibitory effect. For example, the percentage of GFP-positive cells in the miR-511 (or miR-1297)-treated culture was 29.7% (or 25.8%), much lower than NC control culture (40.8%) (Figure 3B), the other miRNAs (miR-26a, miR-125a, miR-132) did not inhibit GFP expression appreciably compared with the control cultures (data not shown). When we mutated the seed sequences of miR-511/1297, the expression of GFP was not decreased obviously in mut-miR-511- or mut-miR-1297-treated cells compared with miR-511- or miR-1297-treated cells (Figure S1).

### TRIB2 expression was suppressed by miR-511/1297

**Figure 4:**
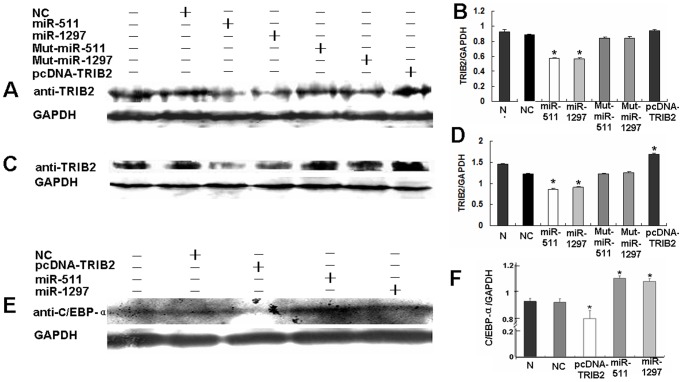
Detection of protein by western blotting. (**A, B**) lung adenocarcinoma A549 cells were treated with miRNAs and their controls, TRIB2 expression was detected and the results showed that its expression in the miR-511- and miR-1297-treated cultures was much lower than that of NC-(or mutation miRNA)-treated cultures (*p<0.01). Relative values for TRIB2 vs GAPDH are indicated to the right of the gel (Fig. 4B). (**C, D**) Another lung adenocarcinoma LTEP-a-2 cells were treated with miRNAs and their controls, TRIB2 expression was aso much lower in the miR-511- and miR-1297-treated cells than that of NC-(or mutation miRNA)-treated cultures (*p<0.01). Relative values for TRIB2 vs GAPDH are shown to the right of the gel (Fig. 4D). (**E, F**) C/EBPα expression was analyzed and the results showed that its expression was increased in the miR-511- and miR-1297-treated cells than that of the control cells (NC group, *p<0.05). Relative values for TRIB2 vs GAPDH are indicated to the right of the gel (Fig. 4F). N, negative control cells. NC, miR-511, miR-1297, mut-miR-511, mut-miR-1297, and pcDNA-TRIB2, cells treated with NC, miR-511, miR-1297, mut-miR-511, mut-miR-1297, and pcDNA-TRIB2 vector, respectively.

TRIB2 expression was further confirmed by western blotting after miR-511/1297 treatment. The results showed that TRIB2 expression significantly decreased in miR-511/1297-treated cells compared with controls (Figure 4A, B). The expression of TRIB2 in mut-miR-511- or mut-miR-1297-treated cells was not reduced compared with miR-511- or miR-1297-treated cells (Figure 4A, B).

To determine whether miR-511 and 1297 could inhibit TRIB2 expression in another lung adenocarcinoma cell line, we studied the expression of GFP and TRIB2 in LTEP-a-2 cell line after miR-511/1297 treatment. The results showed that the intensities of fluorescence from the miRNA-treated cultures was weaker and the percentage of GFP-positive cells in miR-511 (or miR-1297)-treated cultures was also much lower than the NC-or mut-miRNAs-treated cells (Figure S2), and the TRIB2 expression was significantly decreased in miR-511/1297-treated cultures compared with NC-, mu-miR-511-, or mu-miR-1297-treated cultues (Figure 4C, D).

### miR-511/1297 expressing in lung adenocarcinoma tissue and inhibiting lung adenocarcinoma cell proliferation

**Figure 5:**
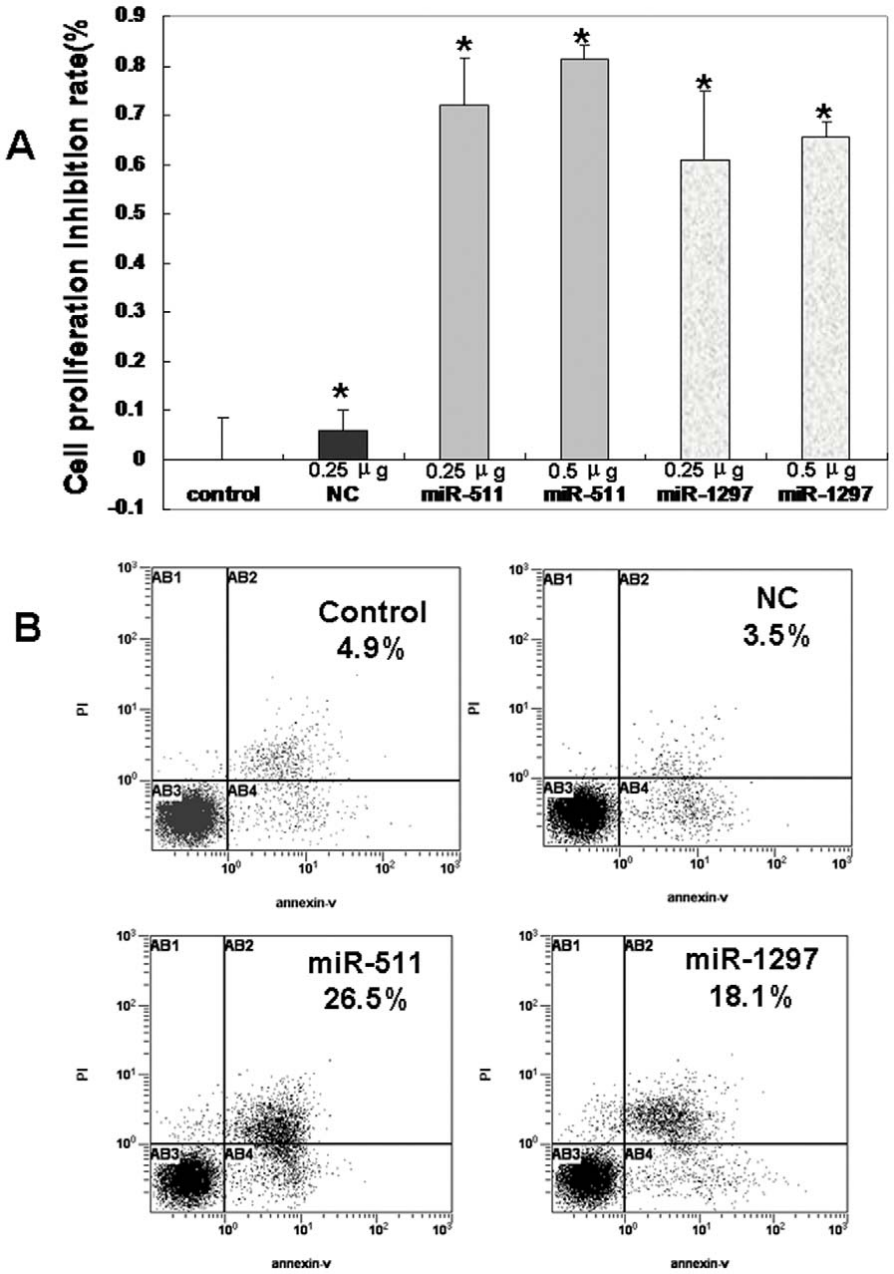
Detection of cell proliferation and apoptosis. (**A**) Proliferation inhibition rate of A549 cells analyzed by the MTT assay. The proliferation inhibition rates of A549 cells were >50% in the miR-511/1297-treated cultures compared with the NC-treated cultures (p<0.05), and the proliferation inhibition rates were dose-dependent, increasing with a higher concentration of miRNAs. (**B**) FACS analysis. Annexin V-FITC/PI staining was performed to evaluate the apoptosis of A549 cells. Apoptotic cells are shown in the upper left, upper right, and lower right quadrants of each panel. Apoptotic cells increased in the miR-511- and miR-1297-treated cells compared with the NC-treated cells.

The above results showed that TRIB2 expression was increased in lung adenocarcinoma. As miRNAs to regulate TRIB2 expression, miR-511 and 1297 were further studied in lung adenocarcinoma tissue by real-time PCR. The expression of miR-511/1297 was found to be lower in carcinoma than its control tissues (Figure 1C), which indicated that these miRNAs might regulate *TRIB2* in lung adenocarcinoma tissue. Thus, we selected A549 cells to study whether these miRNAs could inhibit cell proliferation by suppressing *TRIB2*. After *TRIB2* oncogene expression was inhibited by miR-511/1297, the growth rate of A549 cells was measured by the MTT assay. Our results demonstrated that miR-511/1297 could inhibit A549 cells proliferation obviously compared with NC-treated cultures (*p*<0.05) (Figure 5A). Annexin V-FITC/PI staining results showed little to no detectable apoptosis in the negative and NC-treated cells, while the percentage of apoptotic cells in the miR-511- and miR-1297-treated cultures were 26.5% and 18.1%, respectively, significantly higher than the controls (Figure 5B). Fragmentation and condensation of chromatin were found in A549 cells by the Hoechst staining at 48 h after miR-511/1297 treatment compared with the NC-treated cells (data not shown).

### C/EBPα expression affected by the miR-511/1297 suppression pathway

According to previous studies, the expression of C/EBPα, a transcription factor downstream of TRIB2, can also be regulated by factors that affect TRIB2 expression [13]. Therefore, we examined the expression of C/EBPα by western blot after A549 cells were treated with miR-511/1297. The results showed that C/EBPα was increased in miR-511- and miR-1297-treated cells compared with NC-treated cultures after miR-511/1297 inhibiting TRIB2 expression, while C/EBPα was decreased after over-expression TRIB2 by transfecting pcDNA-TRIB2 vector (Figure 4E, F). Our results showed that miR-511 and miR-1297 could inhibit A549 cell proliferation by downregulation of TRIB2 and upregulation of C/EBPα.

### miR-511/1297 inhibiting lung adenocarcinoma cell proliferation in nude mice

**Figure 6:**
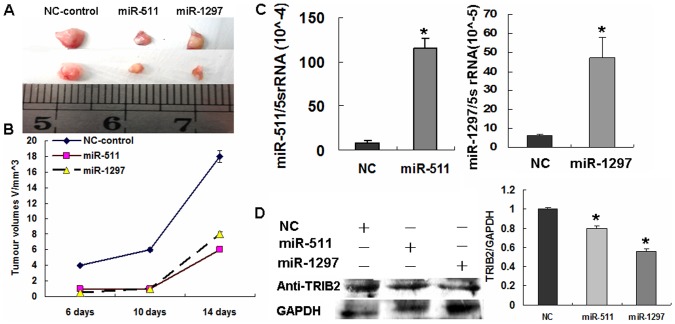
Detection of A549 lung cancer xenografts into nude mice. (**A**) Tumor tissues were removed and measured 15 days after engraftment. (**B**) Growth curve was drawn from measurement taken 6 days after xenograft (n = 4 each group). From A and B, A549 cell xenografts were inhibited and smaller tumor volumes were detected in miR-511- and miR-1297-treated tumors compared with the NC-treated tumors. (**C**) Real-time PCR detection. After isolation of miRNAs from xenografts, higher miR-511 and miR-1297 levels were found in the miR-511- and miR-1297-treated xenografts than controls. (**D**) Western blotting detection. TRIB2 expression was detected in xenografts and the results showed that its expression in the miR-511- and miR-1297-treated xenografts was lower than that of NC-treated controls (*p<0.05). Relative values for TRIB2 vs GAPDH are indicated to the right of the gel.

After miR-511/1297 transfection, a xenograft of A549 cells was subcutaneously injected into the dorsal flank of nude mice. Tumor volumes were calculated after the mice developed palpable tumors. The volumes of xenografts were found to be smaller in mice which received miR-511- and miR-1297-treated cells compared with control cells after a period of two weeks (Figure 6 A B). The miR-511 and miR-1297 levels were found to be higher (Figure 6 C) and TRIB2 expression was decreased in miRNA-treated groups (Figure 6 D). These results demonstrated that treatment with miR-511/1297 could also inhibit A549 cell proliferation in vivo by suppressing *TRIB2* oncogene expression.

## Discussion

miRNAs, together with partner proteins, bind to the 3′ UTR region of their specific target mRNA to regulate target genes by degradation of target mRNAs or inhibition of gene expression [15]. We predicted the possible miRNAs (miR-511 and miR-1297, among others) which could possibly targeting *TRIB2* by using microRNA analysis software online. A plasmid, containing the GFP reporter gene followed by the 3′-UTR of *TRIB2*, was constructed to analyze the interaction between *TRIB2* and miR-511/1297. Our results showed that miR-511 and miR-1297 could downregulate the GFP expression compared with NC-treated cells. The expression of *TRIB2* was further found to be suppressed by miR-511 and miR-1297. As the one factor known to be regulated by *TRIB2* [13], C/EBPα expression was increased after miR-511 (or miR-1297) treatment. Our results demonstrate that miR-511 and miR-1297 could inhibit A549 cell proliferation by suppressing TRIB2 expression and thus increasing C/EBPα expression.

Several previous studies showed that *TRIB2* acted as an oncogene and increasing TRIB2 expression led to AML tumorigenesis [13], [14]. The oncogenic role of *TRIB2* was further confirmed in our study, which showed increased levels of TRIB2 in human lung adenocarcinoma tissue. We also showed that inhibition of *TRIB2* expression by its relevant miRNA (miR-511 or miR-1297) could induce tumor cell apoptosis. Tribbles proteins promote ubiquitin-dependent degradation of their target proteins. *TRIB2* promotes the degradation of C/EBPα and C/EBPβ which have been described as transcription factors [13]. The intact C-terminal constitutive photomorphogenesis 1(COP1)-binding site is necessary for *TRIB2* to degrade C/EBPα [20]. Loss of C/EBPα function is associated with myeloid transformation in a variety of murine models and human leukemias [13], [14], [21]. Moreover, C/EBPα functions as a transcription factor in the absence of *TRIB2*. The transcriptional activity is lost when *TRIB2* is present and bound to C/EBPα, possibly because TRIB2-binding prevents C/EBPα from binding to DNA [22].

C/EBPα has been reported to inhibit cell-cycle progression and to regulate differentiation in various cell types [23]. More evidence pointed out C/EBPα-mediated apoptosis in myeloid cell lines 3 days after transfection with C/EBPα. C/EBPα initiates apoptosis in NIH3T3 cells at 4–8 h after C/EBPα treatment and less than 5% of NIH3T3 cells survived by days 4–5 after transfection. The cell-growth inhibition by C/EBPα could cooperated with cyclin D3 in Hep3B2 cells [24]–[26]. C/EBPα was expressed abundantly in the lung, specifically in type II pneumocytes and in cells of the bronchial epithelium [27], [28]. C/EBPα also played an important role in normal lung development and in the maintenance of normal alveolar structure as determined by knocking out the C/EBPα gene in mice [29]. In our study, the relationship between function of C/EBPα and lung adenocarcinoma cell proliferation was investigated. A549 cell proliferation was suppressed and the C/EBPα transcription factor was increased when miR-511/1297 treatment reduced TRIB2 expression. Our results are supported by Grandinetti's study, which demonstrated that *TRIB2* is overexpressed in lung cancer through downregulation of C/EBPα [22], while upregualtion of C/EBPα in lung cancer cells results in obvious inhibition of cell proliferation, suggesting that upon *TRIB2* knockdown, C/EBPα levels increase and drive lung cancer cell differentiation.

miR-511 and miR-1297 are novel miRNAs and their functions in tumor cell proliferation are not clear. Tserel and colleagues reported that the 3′-UTRs of TLR4 I and TLR4 II were miR-511 target sites and that miR-511 knockdown enhanced TLR4 protein levels in differentiating DCs [30]. Downregulation of miR-511 expression was found in ovarian tumor tissues [31]. Research about miR-1297 has not been reported previously. We first investigated this miRNA function in this study, and a novel finding is that the expression of *TRIB2* could be regulated by miR-511 and miR-1297, which also increased C/EBPα expression. In addition, cell apoptosis was prominent in miR-511- (or miR-1297)-treated cells contrasted with the negative control cultures, suggesting that miR-511 and miR-1297 possibly act as tumor suppression miRNAs.

In summary, our results demonstrated that C/EBPα, as the downstream factor of *TRIB2*, was up-regulated after miR-511 (or miR-1297) treatment, and that miR-511 (or miR-1297) acts as a tumor suppressor genes to induce A549 cell apoptosis by targeting the oncogene *TRIB2*. Study of the *TRIB2* oncogene and its related miRNAs miR-511 and miR-1297) may provide new targets for lung cancer therapy.

## Materials and Methods

### Immunohistochemistry

Lung adenocarcinoma tissue samples (obtained from the Affiliated Hospital to Binzhou Medical University after a curative operation, with approval from the Medical Ethics Committee of Binzhou Medical University. Written informed consent of each patient was obstained.) were fixed in 4% paraformaldehyde embedded in paraffin, and sectioned. Sections were deparaffinized and rehydrated in alcohol, incubated in hydrogen peroxide, followed by 10% normal goat serum (Bei Jing Zhong Shan-Golden Bridge Technology CO, LTD, China). Sections were then incubated with anti-*TRIB2* primary antibodies (1∶300, dilution, Santa Cruz Biotechnology, Inc. USA), and were exposed to the biotin-conjugated goat anti-rabbit IgG (1∶300, dilution, Santa Cruz Biotechnology, Inc. USA).

TRIB2 expression was examined under the Olympus BX51 AX-70 microscope (Olympus, Japan). Image analysis was used by the Image-Pro Plus software. Parameters include positive expression area, mean density and integral optical density (IOD). Brown regions represent protein positive expression. Then, the data of each group was analyzed.

### Construction of pcDNA-GFP-TRIB2–3′UTR vector

The relationship between TRIB2–3′-UTR and its targeted miRNAs was predicted using microRNA analysis software online (http://www.microrna.org/microrna/getMirnaForm.do, or http://www.targetscan.org/index.html). These websites provide a comprehensive analysis of the targeting genes of miRNAs. The 3′-UTR (1739 bp) of *TRIB2* gene was cloned by PCR using the following Primers: forward 5′-TGGTGCTAAGGAAGTGTC-3′ and reverse 5′-CTGGTTACGAAGGGTGAA-3′. Amplification conditions were as follows: 5 min initial denaturation at 95°C followed by 28 cycles of 45 sec denaturation at 95°C, 45 sec annealing at 54°C, 2 min elongation at 72°C. The 3′-UTR was cloned into the T vector (Takara Bio Inc, Japan) to construct T-TRIB2-UTR vector. The 3′-UTR of *TRIB2* was cut from T-TRIB2-UTR vector and inserted to the downstream of the GFP gene in the pcDNA-GFP vector (described previously) [32] by K*pn*I/H*ind*III, constructing the pcDNA-GFP-TRIB2–3′UTR vector. The pcDNA-GFP-TRIB2–3′UTR vector was confirmed by restriction endonuclease digestion and by an automated DNA sequencing (Biosune, Shanghai, China, data not shown). TRIB2 cDNA (1129 bp) was cloned using the following Primers: 5′- CACTCGCCAGCGACTCATCTC -3′ and reverse 5′- TGGAACCTGCTAAGTCTCCGTGG-3′. Amplification conditions were as follows: 5 min initial denaturation at 95°C followed by 30 cycles of 45 sec denaturation at 95°C, 30 sec annealing at 55°C, 1 min elongation at 72°C. TRIB2 cDNA was cloned into T vector (Takara Bio Inc, Japan) to construct T-TRIB2-cDNA vector. TRIB2 cDNA was cut from T-TRIB2-cDNA vector and inserted into pcDNA3.1Neo(+) (Invitrogen, USA) vector by B*amH*I/H*ind*III to form pcDNA-TRIB2 vector.

### Cell culture and transfection

Lung adenocarcinoma cells (A549 and LTEP-a-2 cell lines were, obtained from Shanghai Institute of Cell Biology, China) were cultured in 1640 medium (Hyclone, USA) supplemented with 10% fetal calf serum (Hyclone, USA) and 10 U/ml of penicillin-streptomycin (Sigma, USA) at 37°C with 5% CO_2_.

All transfections were carried out in triplicate. For transfection, 1×10^6^ cells were treated with 0.5 µg miRNA and 0.5 µg pcDNA-GFP-TRIB2–3′UTR plasmid in 2.5 µl of lipofectamine 2000 (Invitrogen, USA), according to the manufacturer's instructions. 48 h after transfection, real-time PCR was performed to detect miRNAs in lung adenocarcinoma cells by SuperTaq Polymerase (Takara Bio Inc, Japan) following the manufacturer's instructions. The concentration of miRNAs was assessed using the RG3000 system (Corbett Research, Australia) with the Quantitect SYBR-Green kit (Qiagen, Germany).

### GFP assay

GFP expression was first observed under a fluorescent microscope (LX71, Olympus, Japan), and then the percentage of GFP positive cells was estimated by flow cytometry (FACS, Beckman Coulter, Inc., USA). At 24 h after transfection, the samples (1×10^5^ cells) were centrifuged for 5 min at 500 g and the supernatant was discarded, the pellet was resuspended in 300 µl PBS and analyzed.

### Western blotting

Total protein lysates were prepared and each 35 µg protein sample was loaded onto 10% SDS-PAGE, transferred to PVDF membranes after electrophoresis, and blocked with 7% non-fat milk in TBST [50 mmol/l Tris-HCl (pH 7.6), 150 mmol/l NaCl, 0.1% Tween-20] for 1.5 h at room temperature. The TBST buffer solution was used to wash the membranes 3 times before immunoblotting using antibody specific for polyclonal rabbit anti-human *TRIB2* (Santa Cruz Biotechnology, Inc. USA) or C/EBP-α at 1∶400 (Bio-world, USA). After being incubation at 4°C overnight and a wash with TBST, the membranes were incubated with a HRP-labeled goat anti-rabbit IgG (1∶5000, Beijing Zhong Shan-Golden Bridge Technology Co., Ltd, China) for 1.5 h at room temperature. The membranes were washed with TBST, then incubated with ECL reagent and exposed. The same membrane also was stripped and re-probed with GAPDH antibody (1∶500, Beijing Zhong Shan-Golden Bridge Technology Co., Ltd, China) as the loading control.

### MTT assay

Considering the oncogenic role of TRIB2, we investigated A549 cell proliferation after miR-511 and miR-1297 treatment by using the MTT assay(3-(4,5-dimethylthiazol-2-yl)-2,5-diphenyltetrazolium bromide, 5 mg/ml, Sigma, USA). A549 cells seeded on 96-well plates (1×10^4^ cells/well) were treated with miRNAs. Each treatment was replicated 3 times on the plate. After 48 h treatment, 10 µl MTT were added to each well to reveal cell proliferation. Cell proliferation inhibition rate = (ODcontrol - ODsample)/ODcontrol×100 (%).

### Annexin V-FITC/PI staining

A549 cells were also collected and stained using Annexin V-FITC/PI apoptosis detection kit. A549 cells were trypsinized and gently washed with serum-containing medium. The samples (1×10^5^ cells) were centrifuged for 5 minutes at 400×g and the supernatant was discarded. The cells were then stained using Annexin V-FITC/PI apoptosis detection kit (KeyGen BioTECH) following the manufacturer's instruction. After incubation at room temperature for 15 min, the apoptotic cells were immediately analyzed by flow cytometry.

### Hoechst staining

Hoechst staining was employed to evaluate cell apoptosis 48 h after being treated with miRNAs. Then, stained with Hoechst 33258 (10 µg/ml) for 10 min. After being washed with PBS, cells were observed using a fluorescence microscope (Olympus, Japan). Each experiment was performed in triplicate.

### A549 lung cancer xenografts

BALB/C-nu mice (nude mice) were obtained from HFK Bio-Technology Co. Ltd, Beijing, China. Animal protocols were approved by the Committee on the Ethics of Animal Experiments of Binzhou Medical University.

48 h after miR-511/1297 treatment, A549 cells (1×10^7^) were trypsinized, counted, gently washed with PBS and subcutaneously injected into the dorsal flank of 6–8-week-old BALB/C-nu mice. Once the mice developed palpable tumors, caliper measurements were taken to measure the tumor volume. Only one person measured the tumors in the experiments in order to prevent observation differences. The tumors were measured between the skin surface layers. The length and width were measured with an accuracy of 0.01 mm. A growth curve was drawn to reflect the tumor growth. Tumor volume was calculated using the formula: v = length×width^2^/2 (length>width) [33], [34].

### Statistical analysis

SAS software was used to analyze the significance of all results. The Student's t-test was used for inter-group comparison. A p-value <0.05 was considered significant.

## Supporting Information

Figure S1 **GFP expression in A549 cells was detected by fluorescence microscopy and FACS.** (A, B) Cells treated with miR-1297 and its mutation miRNA. Fluorescence microscopy: Upper panel, phase-contrast view under visible light. Lower panel, fluorescence to reveal expression of GFP-positive cells. Scale bar = 100 µm. The intensity of GFP expression was weaker and the number of GFP-positive cells was fewer in miR-1297-treated cells than mutation control cultures (Figure S1 A). FACS results showed that the ratio of GFP-positive cells in miR-1297-treated cultures was much lower than that of mut-miR-1297-treated cells (Figure S1 B). (C, D) Cells treated with miR-511 and its mutation miRNA. Fluorescence microscopy: Upper panel, phase-contrast view under visible light. Lower panel, fluorescence to reveal expression of GFP-positive cells. Scale bar = 100 µm. The intensity of GFP expression was aslo weaker and the number of GFP-positive cells was fewer in miR-511-treated cells than mut-miR-511 control (Figure S1 C). The ratio of GFP-positive cells in miR-511-treated cultures was lower than that of mutation control (Figure S1 D).
(DOC)

Figure S2 **GFP expression in LTEP-a-2 cells was detected by fluorescence microscopy and FACS.** (A, B) Cells treated with miR-1297 and its mutation miRNA. Fluorescence microscopy: Upper panel, phase-contrast view under visible light. Lower panel, fluorescence to reveal expression of GFP-positive cells. Scale bar = 100 µm. The intensity of GFP expression was weaker and the number of GFP-positive cells was fewer in miR-1297-treated cells than mutation control (Figure S2 A). The percentage of GFP-positive cells in miR-1297-treated cultures was much lower than that of mutation control (Figure S2 B). (C, D) Cells treated with miR-511 and its mutation miRNA. Fluorescence microscopy: Upper panel, phase-contrast view under visible light. Lower panel, fluorescence to reveal expression of GFP-positive cells. Scale bar = 100 µm. The intensity of GFP expression was weaker and the number of GFP-positive cells was fewer in miR-511-treated cells than mut-miR-511 control (Figure S2 C). The percentage of GFP-positive cells in miR-511-treated cultures was lower than mutation control (Figure S2 D).
(DOC)
