# Supplementary material for: miR-511 and miR-1297 Inhibit Human Lung Adenocarcinoma Cell Proliferation by Targeting Oncogene TRIB2
Source: PLoS One. 2012 Oct 5;7(10):e46090. doi: 10.1371/journal.pone.0046090 (PMC3465292; doi:10.1371/journal.pone.0046090)
Supplement: Figure S1 — GFP expression in A549 cells was detected by fluorescence microscopy and FACS. (A, B) Cells treated with miR-1297 and its mutation miRNA. Fluorescence microscopy: Upper panel, phase-contrast view under visible light. Lower panel, fluorescence to reveal expression of GFP-positive cells. Scale bar = 100 µm. The intensity of GFP expression was weaker and the number of GFP-positive cells was fewer in miR-1297-treated cells than mutation control cultures (Figure S1 A). FACS results showed that the ratio of GFP-positive cells in miR-1297-treated cultures was much lower than that of mut-miR-1297-treated cells (Figure S1 B). (C, D) Cells treated with miR-511 and its mutation miRNA. Fluorescence microscopy: Upper panel, phase-contrast view under visible light. Lower panel, fluorescence to reveal expression of GFP-positive cells. Scale bar = 100 µm. The intensity of GFP expression was aslo weaker and the number of GFP-positive cells was fewer in miR-511-treated cells than mut-miR-511 control (Figure S1 C). The ratio of GFP-positive cells in miR-511-treated cultures was lower than that of mutation control (Figure S1 D). (DOC) [file pone.0046090.s001.doc]

**Figure S1**


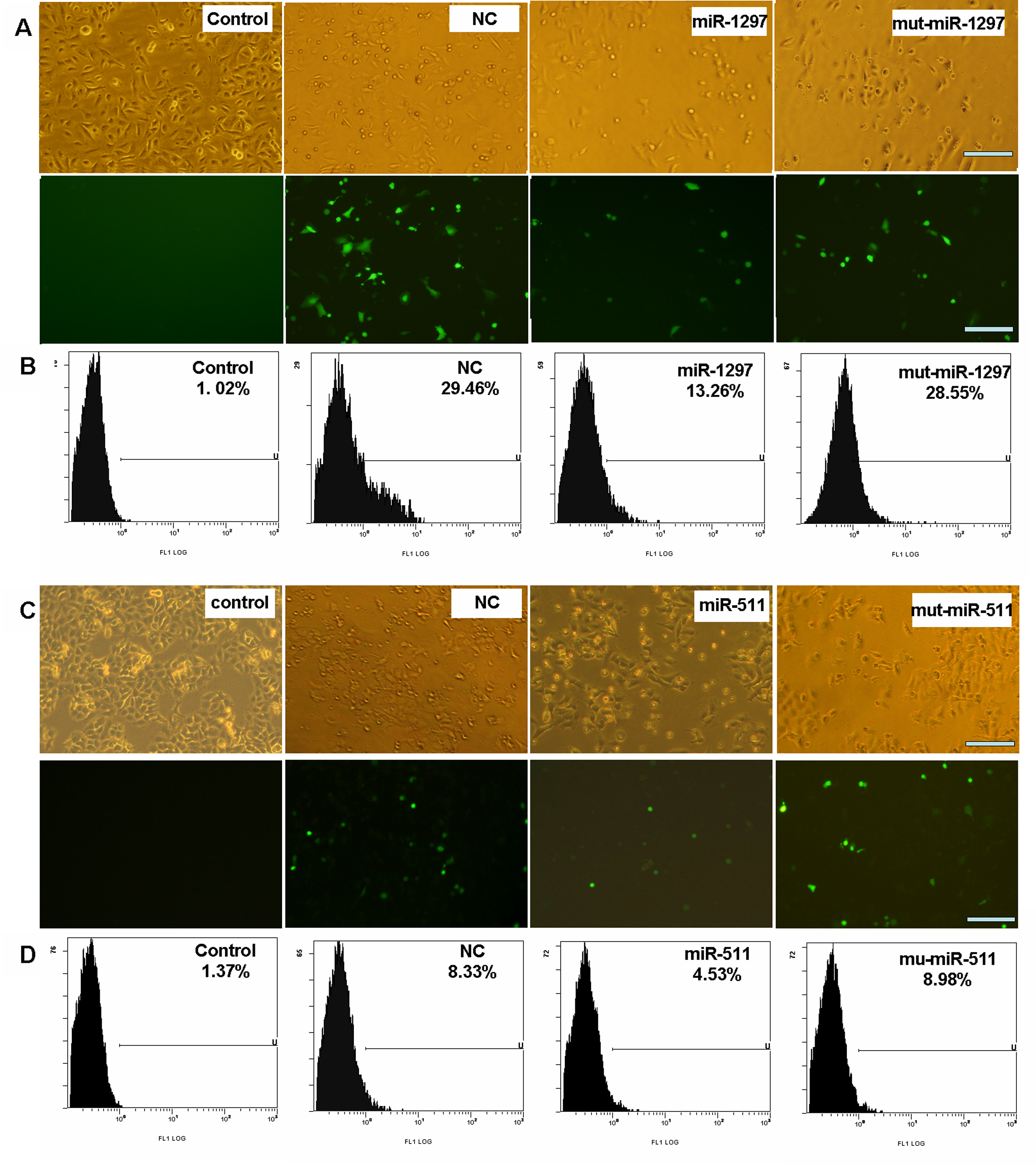


**Figure S1. GFP expression in A549 cells was detected by fluorescence microscopy and FACS.**

**(A, B) Cells treated with miR-1297 and its mutation miRNA.** Fluorescence microscopy: Upper panel, phase-contrast view under visible light. Lower panel, fluorescence to reveal expression of GFP-positive cells. Scale bar=100μm. The intensity of GFP expression was weaker and the number of GFP-positive cells was fewer in miR-1297-treated cells than mutation control cultures (**Figure S1** A). FACS results showed that the ratio of GFP-positive cells in miR-1297-treated cultures was much lower than that of mut-miR-1297-treated cells (**Figure S1** B).

**(C, D) Cells treated with miR-511 and its mutation miRNA.** Fluorescence microscopy: Upper panel, phase-contrast view under visible light. Lower panel, fluorescence to reveal expression of GFP-positive cells. Scale bar=100μm. The intensity of GFP expression was aslo weaker and the number of GFP-positive cells was fewer in miR-511-treated cells than mut-miR-511 control (**Figure S1** C). The ratio of GFP-positive cells in miR-511-treated cultures was lower than that of mutation control (**Figure S1** D).
