# Supplementary material for: miR-511 and miR-1297 Inhibit Human Lung Adenocarcinoma Cell Proliferation by Targeting Oncogene TRIB2
Source: PLoS One. 2012 Oct 5;7(10):e46090. doi: 10.1371/journal.pone.0046090 (PMC3465292; doi:10.1371/journal.pone.0046090)
Supplement: Figure S2 — GFP expression in LTEP-a-2 cells was detected by fluorescence microscopy and FACS. (A, B) Cells treated with miR-1297 and its mutation miRNA. Fluorescence microscopy: Upper panel, phase-contrast view under visible light. Lower panel, fluorescence to reveal expression of GFP-positive cells. Scale bar = 100 µm. The intensity of GFP expression was weaker and the number of GFP-positive cells was fewer in miR-1297-treated cells than mutation control (Figure S2 A). The percentage of GFP-positive cells in miR-1297-treated cultures was much lower than that of mutation control (Figure S2 B). (C, D) Cells treated with miR-511 and its mutation miRNA. Fluorescence microscopy: Upper panel, phase-contrast view under visible light. Lower panel, fluorescence to reveal expression of GFP-positive cells. Scale bar = 100 µm. The intensity of GFP expression was weaker and the number of GFP-positive cells was fewer in miR-511-treated cells than mut-miR-511 control (Figure S2 C). The percentage of GFP-positive cells in miR-511-treated cultures was lower than mutation control (Figure S2 D). (DOC) [file pone.0046090.s002.doc]

**Figure S2**


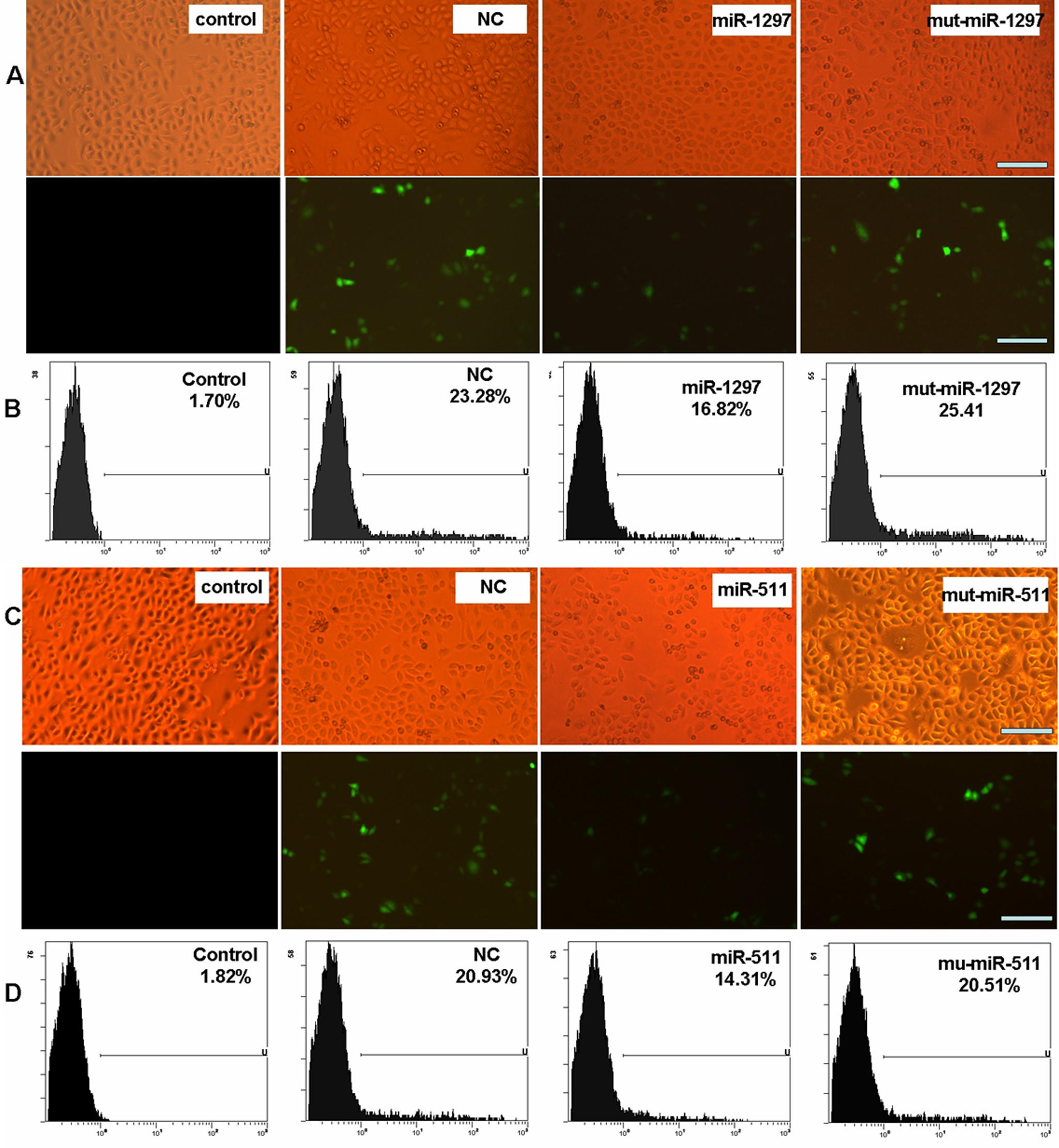


**Figure S2. GFP expression in** [**LTEP-a-2**](http://www.cellbank.org.cn/detail_1.asp?id=118&serial=TCHu 33) **cells was detected by fluorescence microscopy and FACS.**

**(A, B) Cells treated with miR-1297 and its mutation miRNA.** Fluorescence microscopy: Upper panel, phase-contrast view under visible light. Lower panel, fluorescence to reveal expression of GFP-positive cells. Scale bar=100μm. The intensity of GFP expression was weaker and the number of GFP-positive cells was fewer in miR-1297-treated cells than mutation control (**Figure S2** A). The percentage of GFP-positive cells in miR-1297-treated cultures was much lower than that of mutation control (**Figure S2** B).

**(C, D) Cells treated with miR-511 and its mutation miRNA.** Fluorescence microscopy: Upper panel, phase-contrast view under visible light. Lower panel, fluorescence to reveal expression of GFP-positive cells. Scale bar=100μm. The intensity of GFP expression was weaker and the number of GFP-positive cells was fewer in miR-511-treated cells than mut-miR-511 control (**Figure S2** C). The percentage of GFP-positive cells in miR-511-treated cultures was lower than mutation control (**Figure S2** D).
